# Ablation of Neuropilin 1 from glioma-associated microglia and macrophages slows tumor progression

**DOI:** 10.18632/oncotarget.6877

**Published:** 2016-01-10

**Authors:** Jeremy T. Miyauchi, Danling Chen, Matthew Choi, Jillian C. Nissen, Kenneth R. Shroyer, Snezana Djordevic, Ian C. Zachary, David Selwood, Stella E. Tsirka

**Affiliations:** ^1^ Department of Pharmacology, Stony Brook University, Stony Brook, NY, USA; ^2^ Department of Pathology, Stony Brook University, Stony Brook, NY, USA; ^3^ Institute of Structural and Molecular Biology, University College London, London, UK; ^4^ Centre for Cardiovascular Biology and Medicine, Division of Medicine, University College London, London, UK; ^5^ Wolfson Institute for Biomedical Research, University College London, London, UK

**Keywords:** glioma, Neuropilin 1, microglia, immunomodulation, macrophage

## Abstract

Gliomas are the most commonly diagnosed primary tumors of the central nervous system (CNS). Median times of survival are dismal regardless of the treatment approach, underlying the need to develop more effective therapies. Modulation of the immune system is a promising strategy as innate and adaptive immunity play important roles in cancer progression. Glioma associated microglia and macrophages (GAMs) can comprise over 30% of the cells in glioma biopsies. Gliomas secrete cytokines that suppress the anti-tumorigenic properties of GAMs, causing them to secrete factors that support the tumor's spread and growth. Neuropilin 1 (Nrp1) is a transmembrane receptor that in mice both amplifies pro-angiogenic signaling in the tumor microenvironment and affects behavior of innate immune cells. Using a Cre-lox system, we generated mice that lack expression of Nrp1 in GAMs. We demonstrate, using an *in vivo* orthotopic glioma model, that tumors in mice with Nrp1-deficient GAMs exhibit less vascularity, grow at a slower pace, and are populated by increased numbers of anti-tumorigenic GAMs. Moreover, glioma survival times in mice with Nrp1-deficient GAMs were significantly longer. Treating wild-type mice with a small molecule inhibitor of Nrp1's b1 domain, EG00229, which we show here is selective for Nrp1 over Nrp2, yielded an identical outcome. Nrp1-deficient or EG00229-treated wild-type microglia exhibited a shift towards anti-tumorigenicity as evident by altered inflammatory marker profiles *in vivo* and decreased SMAD2/3 activation when conditioned in the presence of glioma-derived factors. These results provide support for the proposal that pharmacological inhibition of Nrp1 constitutes a potential strategy for suppressing glioma progression.

## INTRODUCTION

Gliomas, the most common primary malignancies of the central nervous system (CNS), are diagnosed at a rate of 17,000 new cases per year in the United States [1]. Median time of survival after diagnosis, regardless of clinical management strategy, is dismal, averaging between 12 to 15 months [2]. Current treatment modalities consist of concomitant radio- and chemotherapy but are suboptimal in slowing disease progression. Patients incur frequent complications including seizures, fluctuating neurological symptoms, and adverse effects of chemotherapy. Thus, there is a substantial need to identify more effective and specific pharmacological targets for treatment. Apart from cytotoxic and anti-angiogenic therapies, modulation of the immune system is a promising strategy, as innate and adaptive immunity are implicated in playing important roles in cancer progression [3].

Neuropilin 1 (Nrp1), a cell-surface transmembrane receptor that functions as a co-receptor for several signaling pathways, has received significant attention as a potential therapeutic target in glioma as well as in other cancer subtypes. Increased Nrp1 expression in glioma biopsies, specifically in *glioma tumor cells*, correlates with increased malignancy [4], whereas reducing Nrp1 expression in glioma cells suppresses migration [5], proliferation and survival *in vitro*, and stem cell viability and tumor growth *in vivo* [6, 7].

Nrp1 has also been shown to function as a co-receptor for VEGF-R on macrophages in the periphery in mouse tumor models, where it plays a key role in the accumulation of immunosuppressive and pro-angiogenic macrophages at sites of tumor hypoxia in pancreatic adenocarcinoma, breast cancer, and lung carcinoma, [8].

Microglia, the resident macrophages of the CNS, can comprise over 30% of the cells in glioma biopsies [9]. Gliomas are known to secrete cytokines that suppress the anti-tumorigenicity of microglia and other macrophages, causing them in turn to secrete factors that support tumor growth and spread [10]. However, it is also known that these glioma-associated microglia and macrophages (GAMs) can undergo an anti-tumorigenic shift depending on the microenvironment and the cytokines they are exposed to [11, 12]. Gliomas have been shown to express high levels of TGF-β, VEGFA, HGF, and Sema3a, which promote tumor growth [13], neo-vascularization [14], invasiveness [15], and dispersal [16]. Important to how the receptors for these cytokines conduct signaling is their co-receptor Neuropilin 1 (Nrp1), which amplifies the activation of their downstream effector pathways [17-22]. These receptors are expressed by macrophages as well [23, 24] and likely have an effect on their activity within tumor micro-environments enriched in their ligands.

Our group has shown that Nrp1 plays an important role in microglial activation and polarization [25]. More specifically, we have shown [25] that pro-tumorigenic M2 polarization of microglia can be blocked by a small molecule inhibitor of Nrp1's b1 domain, EG00229 [26]. The b1 domain binds and amplifies signaling through the VEGF-A and TGFβ pathways ([26, 27]). Based on the above information, our group hypothesized that genetic ablation of Nrp1 from microglia and macrophages populating the glioma microenvironment would lead to a decrease in the capacity of the microenvironment to promote their pro-tumorigenic polarization and thus result in reduced tumor growth.

We demonstrate here using a syngeneic glioma model that tumors develop more slowly in mice in which Nrp1 has been genetically ablated from their microglia and macrophages. This decrease in tumor growth is correlated with an increase in GAM density, decreased neovascularization, and increased anti-tumorigenic polarization of the GAMs populating the tumors. In line with these findings, we find similar disease outcomes in mice administered an inhibitor of Nrp1's b1 domain. Our findings support the proposal that targeting Nrp1 may be a promising therapeutic strategy for malignant glioma through the promotion of anti-tumorigenic polarization of GAMs, in addition to suppressing Nrp1's previously characterized roles in amplifying angiogenic and proliferative signaling in endothelial and glioma-derived cancer cells, respectively.

## RESULTS

### Glioma associated microglia and macrophages express Nrp1

**Figure 1:**
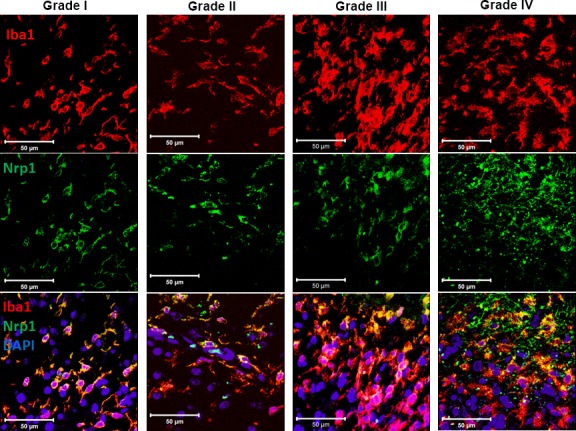
Expression of Nrp1 by glioma-associated microglia/macrophages in human tumor biopsies Representative images of biopsies from patients diagnosed with a pilocytic astrocytoma (grade I), grade II glioma, grade III glioma, and grade IV glioblastoma multiforme (from left to right), which were stained for the microglial/macrophage marker Iba1 (red), Nrp1 (green), and DAPI (Blue). White bars, 50μm.

A critical factor in the progression of gliomas is their interaction with the surrounding tissue and cells. The tumor microenvironment directs and modulates the growth and metastatic propensity of glioma cells [34]. It has been shown that Nrp1 expression by tumor cells is correlated with the degree of biologic aggression of human gliomas [4, 20] but it is not known whether Nrp1 is also expressed by macrophages within the human tumor microenvironment. We thus investigated whether glioma-associated microglia and macrophages (GAMs) express Nrp1 using human tumor biopsies. Nrp1 expression was detected by immunofluorescence in Iba1-positive GAMs in glioma biopsies of varying grades (Figure 1).

### Glioma growth is impaired in mice with Nrp1-deficient microglia/macrophages and mice treated with a pharmacological inhibitor of Nrp1, EG00229

Since genetic depletion of Nrp1 from tumor-associated macrophages has been reported to decrease tumor growth in the periphery for pancreatic and lung tumor xenografts in mice [8], we next investigated whether CNS glioma tumor burden is different in wild type (wt) mice compared to mice with Nrp1-deficient microglia and macrophages. We generated mice that constitutively lack Nrp1 expression in microglia and macrophages (Nrp1^MgKO^) by crossing mice with lox-p sites flanking exon 2 of the Nrp1 gene (Nrp1^fl/fl)^ to mice expressing Cre recombinase under the Csf1R promoter [30, 31]. For the disease model, we used the GL261 glioma cell line, which was derived from a chemically-induced C57BL/6 murine astrocytoma [28] and bears mutations in the tumor suppressor p53 and the oncogene KRas [35]. We previously stably-transfected this cell line with eGFP, generating GL261-eGFP, which was characterized in culture and in an *in vivo* glioma model [29]. Nrp1 mRNA expression was detected in the GL261-eGFP cell line and in wt microglia using primers designed to amplify a region of exon 2 that is excised by Cre recombination, but undetectable in Nrp1^MgKO^ microglia (Supplementary Figure 1). Additionally, Csf1R expression relative to Iba1 expression, in the CNS of wild type and Nrp1^MgKO^ mice remained unchanged (Supplementary Figure 1)

**Figure 2:**
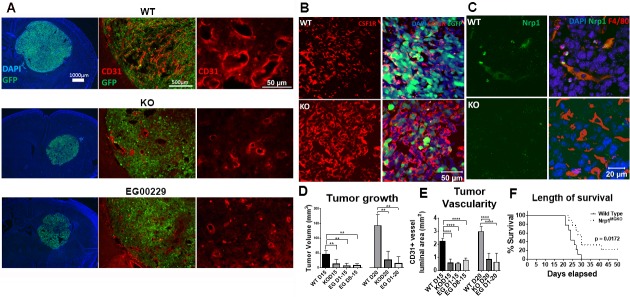
Mice lacking Nrp1 in microglia/macrophages and mice treated with an Nrp1 inhibitor exhibit impaired glioma growth **A.** Representative images of the GL261-eGFP- gliomas and CD31^+^ tumor vasculature in wild-type (wt) mice (top), Nrp1^MgKO^ mice (middle), and wt mice administered the Nrp1 inhibitor EG00229 (75μM) via mini-osmotic pump/cannula (bottom) at 15 days post-glioma induction (PGI). **B.** Representative staining of Csf1R in tumors from wt and Nrp1^MgKO^ mice. **C.** Representative staining of Nrp1 by F4/80+ GAMs in wt and Nrp1^MgKO^ mice. **D.** Quantification of tumor areas, and **E.** quantification of vessel luminal areas bordered by CD31^+^ staining in gliomas in wt, Nrp1^MgKO^, and EG00229-treated mice at 15 and 20 days PGI. Mice were administered EG00229 either throughout the disease course or after day 8 (D8, **= *p* < 0.01, ****= *p* < 0.0001). **F.** Survival curve post-glioma induction in wt and Nrp1^MgKO^ mice. Two Nrp1^MgKO^ mice survived to day 50, at which point the experiment was terminated (WT: *n* = 9, KO: *n* = 9).

Gliomas were introduced into wt and Nrp1^MgKO^ mice and allowed to engraft and develop. At 15 and 20 days after tumor initiation, tumor volumes were significantly lower in the Nrp1^MgKO^ mice (Figure 2A, left column; D). Blood vessel density was visualized using an anti-CD31 antibody and vessel luminal areas summated and quantified from random points throughout the tumors. Gliomas in Nrp1^MgKO^ were found to have significantly decreased blood vessel luminal area relative to tumors in wt mice (Figure 2A middle and right columns, E). Additionally, tumor tissue from both genotypes was populated by prominent numbers of Csf1R expressing cells (Figure 2B). F4/80 GAMs in tumor tissue of wt mice stained positive for Nrp1 expression, while Nrp1 was not detectable in GAMs of Nrp1^MgKO^ mice (Figure 2C).

To examine whether Nrp1 deficiency in GAMs had an effect on survival time, tumors were allowed to engraft in wt and Nrp1^MgKO^ mice and disease outcome was tracked until the terminal stages of the disease. All mice within the wt group were euthanized by 30 days PGI due to ethical concerns, whereas two mice from the Nrp1^MgKO^ group survived until day 50, at which point they were euthanized (Figure 2F). Nrp1^MgKO^ mice displayed a significant increase in survival time based on a Mantel-Cox log rank test (*p* = 0.0172, Figure 2F).

We then investigated whether pharmacologic inhibition of Nrp1 in wild-type mice with EG00229, which blocks the b1 domain of Nrp1, would have similar effects on tumor growth [26]. Pharmacokinetic evaluation of EG00229 in mice revealed a relatively short half-life of 35 min (Supplementary Figure 2), suggesting that focal delivery via osmotic mini-pump would be the preferred route of delivery. We established that intraperitoneal administration of EG00229 at a concentration of 75 μM had no overt adverse effects in mice (data not shown) and used this concentration for loading the osmotic mini-pumps.

Gliomas were introduced in wt mice and EG00229 delivered directly to the glioma injection site via a cannula connected to the pump for 15 or 20 days. Cannulas delivered the drug at a rate of 9.337μg dissolved in 0.25μL vehicle/hour to an estimated volume of distribution of approximately 20mm^3^, based off similar studies [36]. Due to these measurements, we estimated the drug's resulting concentration in the tumor tissue to be approximately 1 μM. We established that mice treated with saline had similar tumor burden to wt mice (Supplementary Figure 3). Gliomas were found to be significantly smaller and have decreased CD31^+^ vessel luminal area in mice administered EG00229 relative to those that developed in control wt mice (Figure 2A, 2D, 2E). To investigate whether delayed administration of the drug could still affect tumor volume, tumor development and vessel luminal area were assessed and found to still be significantly reduced at 15 days-post glioma induction (PGI) when EG00229 was administered 8 days PGI (Figure 2D, 2E). No significant difference in tumor growth or vascularity was observed between tumors from Nrp1^MgKO^ mice or EG00229-treated wt mice at any time point (Figure 2D, 2E).

### EG00229 binds specifically to Nrp1

**Figure 3:**
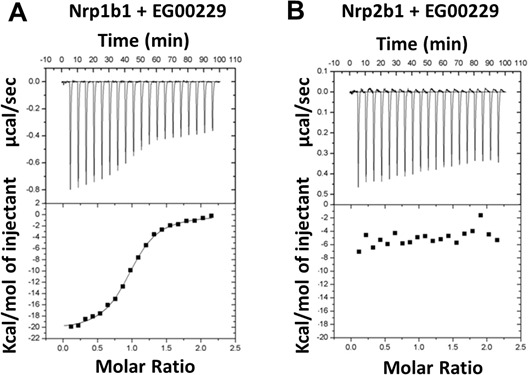
Interaction of EG00229 with recombinant Nrp1b1 and Nrp2b1 domain measured by ITC Representative experiment carried out by titrating EG00229 into **A.** Nrp1b1 and **B.** Nrp2b1. Titration data and integrated heat measurements are shown in the upper and lower plots, respectively.

The other Neuropilin isoform, Nrp2, is structurally similar to Nrp1 and has signaling roles in the CNS and other organ systems that partially overlap but are also distinct from those of Nrp1. To assess the selectivity of EG00229 towards Nrp1, isothermal calorimetry (ITC) was performed. EG00229 exhibited clear binding to a single site on Nrp1b1 domain with associated Kd of 0.2 μM, ΔH = −20640 μCal/mol and ΔS = −42.4 (μCal/mol)/K (Figure 3A). In contrast, interaction between EG00229 and the Nrp2b1 domain was not detected (Figure 3B), indicating that EG00229 specifically interacts with Nrp1.

### Gliomas in mice with Nrp1-deficient microglia/macrophages and mice treated with EG00229 exhibit increased microglial/macrophage infiltration

**Figure 4:**
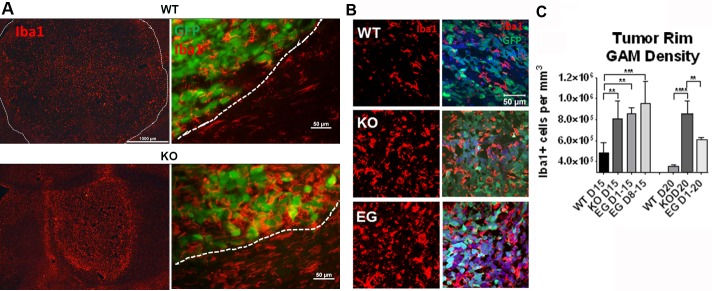
Nrp1 ablation or inhibition in microglia/macrophages increases their density within the glioma border **A.** Representative images of Iba1^+^ microglia/macrophages (red) in gliomas (white dotted lines demarcate borders) in wt (top) and Nrp1^MgKO^ mice (bottom) at 15 days PGI. **B.** Representative images of microglia/macrophage staining (Iba1, red) near the tumor border in wt mice, Nrp1^MgKO^ mice, and wt mice treated with EG00229 for 15 days. Tumor cells are GFP+ (green). **C.** Quantification of the average density of Iba1+ microglia/macrophages within the tumor borders of wt mice, Nrp1^MgKO^ mice, and mice treated with EG00229 for 15 days (D1-15), days 8-15 of the disease (D8-15), or 20 days (D1-20, **= *p* < 0.01, ***= *p* < 0.001, ****= *p* < 0.0001); WT D15, WT D20, KO D20: *n* = 5; KO D15: *n* = 6, EGD1-15, EG D1-20: *n* = 3; EG D8-15: *n* = 4).

Although pharmacologic inhibition of Nrp1 also targets tumor- and endothelial-Nrp1, genetic ablation of Nrp1 solely from microglia and macrophages decreased tumor volume and vascularity to the extent seen with the pharmacological treatment (Figure 2), suggesting that a critical role for Nrp1 in this setting is via signaling to/from the GAMs. To investigate whether ablating Nrp1 expression in GAMs alters their effect on the tumor or the tumor environment, tumor sections were immunostained using the microglia/macrophage marker, Iba1. Gliomas in wt mice were characterized by an Iba1^+^ cell density that was relatively uniform throughout the tumor (Figure 4A). In contrast, gliomas in Nrp1^MgKO^ mice were encompassed by a dense layer of Iba1^+^ cells not observed in the wt mice (Figure 4A). Immunoblotting of crude tumor lysates yielded similar results: higher levels of the microglia/macrophage marker F4/80 were present in Nrp1^MgKO^ tumor lysates, accompanied by increased expression of TSPO, a marker of neuro-inflammation (Supplementary Figure 4). GAM density was similarly increased in the rims of tumors from Nrp1^MgKO^ mice at 15 and 20 days of the disease course relative to GAM density in wt mice (Figure 4). Additionally, GAM density was found to be significantly elevated in the core of tumors in Nrp1^MgKO^ mice although the difference was most profound at the rim (Supplementary Figure 4). GAM density within the tumor rims of EG00229-treated wt mice was, likewise, significantly elevated relative to the density of GAMs in the tumor rim in untreated wt mice (Figure 4B, 4C) and similar to that observed in the Nrp1^MgKO^ mice at 15 days PG1 but not quite as high at 20 days PGI (Figure 4C).

### Nrp1-deficient microglia/macrophages and those in EG00229 treated mice exhibit increased anti-tumorigenic polarization

**Figure 5:**
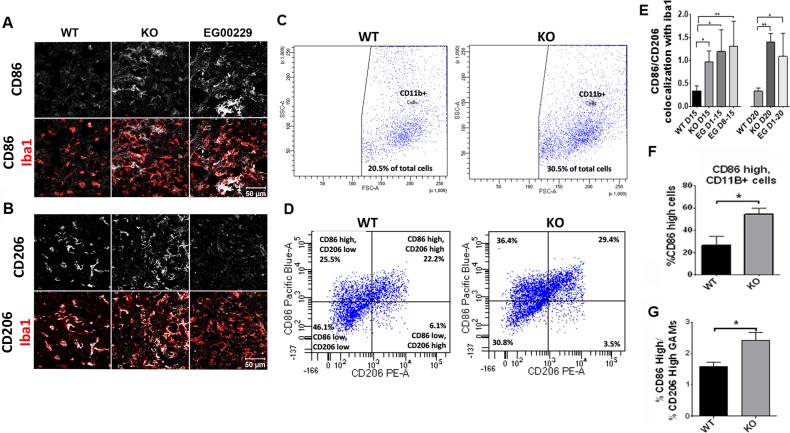
Nrp1 ablation or inhibition in microglia and macrophages alters their polarization in gliomas **A.**-**B.** Representative images of CD86 (A) and CD206 (B) staining (white) in Iba1^+^ (red) microglia/macrophages in gliomas from wt mice, Nrp1^MgKO^ mice, and wt mice treated with EG00229 for 15 days. **C.** CD11b+ cell populations isolated from whole tumor homogenate of wt and Nrp1^MgKO^ mice (vertical axis: side scatter, bottom axis: forward scatter) **D.** Expression of CD86 and CD206 from CD11b-gated macrophages isolated from wt and Nrp1^MgKO^ whole tumor homogenate. **E.** Quantification of co-localization ratio between CD86 and Iba1 to the co-localization of CD206 and Iba1 in microglia/macrophages within the tumor borders of wt mice, Nrp1^MgKO^ mice, and mice treated with EG00229 (*= *p* < 0.05, **= *p* < 0.01; WT D15, WT D20: *n* = 4; KO D15, KO D20, EGD1-15, EG D1-20: *n* = 3; EG D8-15: *n* = 5). **F.** Total percentage of CD11b+ cells which had high expression of CD86 in wt and Nrp1^MgKO^ whole tumor homogenate and **G.** the ratios of CD86 high expressing cells to CD206 high expressing cells in wt and Nrp1^MgKO^ whole tumor homogenate (*= *p* < 0.05, WT: *n* = 4, KO: *n* = 5).

Since macrophages are frequently polarized to a pro-tumorigenic phenotype that supports tumor vascularization and growth, we sought to understand how an increase in GAM density surrounding and in tumors of Nrp1^MgKO^ and EG00229-treated wt mice could be inversely correlated with these outcomes. As Nrp1-dependent pathways can play a role in the behavior of tumor-associated macrophages in the periphery, we examined the phenotypic state of the GAMs by assessing them for expression of CD86, CD206, and TSPO, which are markers of inflammatory status. Relative expression of these markers was quantified by calculating co-localization between the markers and either F4/80 or Iba1 as markers of the GAMs. The ratio of CD86 to CD206 is used as an indicator of the ratio of M1 (inflammatory, anti-tumorigenic) polarization to M2 (pro-tumorigenic, anti-inflammatory) polarization in populations of microglia and macrophages [37]. This ratio was significantly higher (i.e., shifted towards M1) in populations of GAMs populating tumor rims in Nrp1^MgKO^ mice at days 15 and 20 days PGI relative to wt mice at the same time points (Figure 5). Additionally, the percentage of CD11b+ GAMs expressing high levels of CD86 and the ratio of GAMs expressing high levels of CD86 relative to those expressing high levels of CD206 were significantly higher in whole tumor extract of Nrp1^MgKO^ mice (Figure 5). Expression of TSPO, a general marker for inflammatory activation, was significantly elevated in GAMs of Nrp1^MgKO^ mice relative to those of wt mice at 15 days PGI (Supplementary Figure 4). GAMs from tumors in wt mice treated with EG00229 for variable amounts of time were similarly found to have an elevated ratio of CD86 to CD206 expression in comparison to GAMs populating tumors in untreated mice (Figure 5). Additionally, TSPO expression in GAMs was significantly higher in gliomas in mice treated with EG00229 relative to wt mice (Supplementary Figure 4).

### Nrp1 depletion or pharmacologic inhibition in microglia alters their association with glioma-derived cells and increases their anti-tumorigenic polarization in the presence of glioma-derived cytokines

Since inhibition of Nrp1-dependent pathways has been reported to affect the growth of glioma-derived tumor cell lines [7, 20], we investigated the capacity of increasing amounts of EG00229 to suppress growth and migratory behavior of the GL261-eGFP cell line *in vitro*. Significant effects on cell survival (as assayed using propidium iodide (PI) staining) and migration were first seen at 12.5μM (Supplementary Figure 5). As we estimated steady state concentrations of EG00229 administered to mice to be approximately 1μM, we do not expect that this was a significant factor affecting tumor growth in those mice. However, its contribution to the disease course cannot be ruled out based on our data.

**Figure 6:**
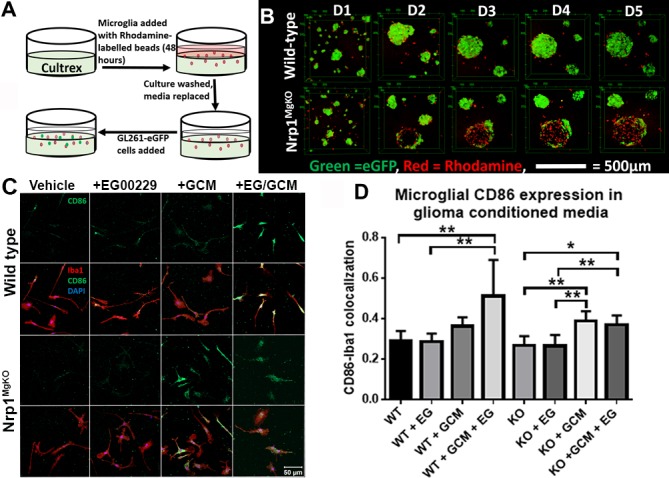
Nrp1-deficient microglia exhibit a different pattern of association and polarization in the presence of glioma-derived cells and factors **A.** Experimental setup for culturing primary microglia and GL261-eGFP cells in cultrex basement membrane extract. **B.** Representative images of microglia-GL261-eGFP associations over 5 days in cultrex basement membrane extract. **C.** Representative images of CD86 expression (green) by Iba1-stained wt and Nrp1^MgKO^ microglia (red) pre-treated with DMSO/vehicle or 12.5μM EG00229 (EG) for 12 hours, followed by exposure to glioma-conditioned media (GCM) for 12 hours. **D.** Quantification of CD86-Iba1 co-localization in EG00229- and GCM-treated microglia (*= *p* < 0.05, **= *p* < 0.01, all groups: *n* = 3).

Having noted differences in the polarity of wt and Nrp1^MgKO^ GAMs *in vivo*, we investigated whether microglia and GL261-eGFP interact differently *in vitro* depending on the presence or absence of Nrp1 signaling by microglia. GL261-eGFP cells were plated onto basement membrane extract in the presence of wt or Nrp1^MgKO^ microglia and they were grown together for 5 days (Figure 6A). GL261eGFP cells assembled into spheres while nearby microglia were attracted into these spheres (Figure 6B). Notably, as Nrp1^MgKO^ microglia congregated around GL261-eGFP spheres, the fluorescence detected from the GL261-eGFP cells populating the spheres decreased. This was not observed when wt microglia migrated into GL261-eGFP spheres (Figure 6B), supporting the idea that Nrp1^MgKO^ microglia associate differently with glioma-derived cells.

We previously established that GL261-conditioned media (GCM) alters microglial activity *in vitro* [29]. We examined whether Nrp1 deficiency affects the capacity of GCM to modify the microglial activation state. Wt or Nrp1^MgKO^ microglia, pre-treated with EG00229 or DMSO vehicle, were cultured in the presence of GCM for 12 hours, and CD86 expression was evaluated by immunofluorescence. Wt microglia pre-treated with EG00229 showed a significant increase in the M1 marker, CD86, after GCM exposure, relative to those that were not exposed to GCM, whereas GCM-exposed wt microglia that were not pre-treated with EG00229 failed to show a significant increase in CD86 expression (Figure 6C, 6D). Consistent with this finding, CD86 expression increased with GCM treatment of Nrp1^MgKO^ microglia whether or not they were pre-treated with EG00229 (Figure 6C, 6D).

### Nrp1 depletion or pharmacologic inhibition in microglia impairs the activation of the SMAD2/3 pathway

**Figure 7:**
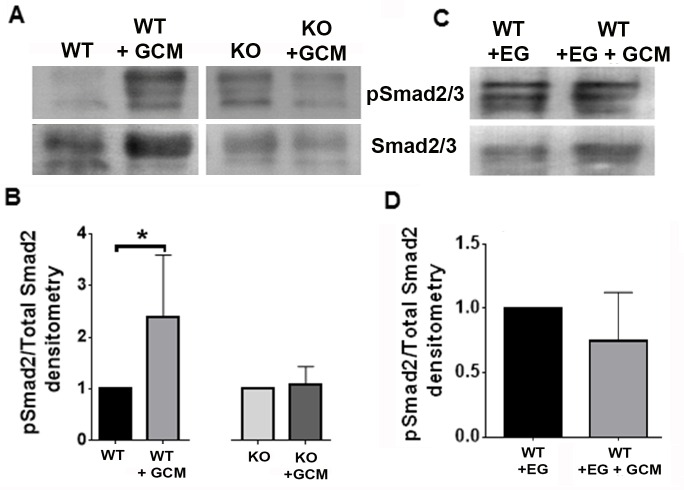
Nrp1 ablation or inhibition blocks SMAD2/3 activation in glioma-conditioned media **A.** Representative blots of pSMAD2/3 and SMAD2/3 expression by wt and Nrp1^MgKO^ microglia after stimulation with GL261-conditioned media (GCM). B. Quantification of SMAD2/3 phosphorylation relative to total SMAD2/3. expression in wt and Nrp1^MgKO^ microglia. GCM-treated samples were standardized to untreated samples (*= *p* < 0.05). **C.**-**D.** Representative blot (C) and quantification (D) of pSMAD2/3 to total SMAD2/3 levels in microglia pre-treated with EG00229 and then either treated with DMEM + 10% FBS (WT+EG) or GCM (WT+EG+GCM). GCM-treated samples were standardized to untreated samples (WT, WT+GCM: *n* = 5, KO, KO+GCM: *n* = 4, WT+EG, WT+EG+GCM: *n* = 5).

To address why Nrp1-deficient and EG00229-treated wt microglia showed differences in M1 polarization in the presence of glioma-derived mediators, we investigated whether they exhibited deficits in signaling via Nrp1-dependent pathways related to polarization. Since Nrp1 is a co-receptor for TGFβ, and having previously demonstrated that microglia show increased activation of the M2-associated SMAD2/3 pathway in the presence of the Nrp1 agonist, tuftsin [25], we investigated whether the activity of this pathway differed between wt and Nrp1^MgKO^ microglia in the presence of GCM. We assessed basal activation of SMAD2/3 in wt and Nrp1^MgKO^ microglia: no appreciable difference in SMAD2/3 expression or its activation was evident between the two cell populations (Supplementary Figure 4). Wt microglia were incubated with or without EG00229 and stimulated with GCM for 30 minutes. Nrp1^MgKO^ microglia were also stimulated with GCM for 30 minutes and SMAD2/3 activation assessed by immunoblotting for the ratio of phosphorylated SMAD2/3 to total SMAD2/3. Wt microglia treated with GCM showed significantly increased SMAD2/3 activation relative to the wt control, whereas the Nrp1^MgKO^ microglia did not (Figure 7A, 7B). Similarly, wt microglia pre-treated with EG00229 did not show activation of SMAD2/3 after treatment with GCM (Figure 7C, 7D).

## DISCUSSION

Our results demonstrate that Nrp1 signaling in GAMs plays a key role in their interaction with the glioma microenvironment, promoting their polarization to a pro-tumorigenic phenotype and thereby facilitating tumor growth and neovascularization. We present evidence that ablation of Nrp1 in GAMs or focal treatment with EG00229, an inhibitor of Nrp1's b1 domain, modulates the way GAMs interact with glioma-derived tumor cells and tumor-cell generated cytokines, resulting in alterations in their polarization which correlate with slower disease progression.

Nrp1 is also expressed by other cell types, raising the question of whether the ability of EG00229 to suppress tumor growth is also due to its effects on endothelial cells [26] and/or the glioma tumor cells. The EG00229 concentrations used *in vivo* were not high enough to be cytotoxic to tumor cells, but other effects on cells within the tumor microenvironment cannot be ruled out in contributing to a slower disease course.

The related receptor Nrp2 is also expressed on microglia. As we presented here, ITC binding using recombinant Nrp1b1 and Nrp2b1 domains demonstrated that EG00229 was fully selective for Nrp1b1, suggesting that it is only necessary to inhibit Nrp1 to achieve anti-tumoral immunity, again decreasing the potential for toxicity at the doses required for therapeutic effect. New and more potent EG00229 analogues are being explored that will improve its utilization in the clinical setting.

Our results demonstrate that Nrp1 ablation from GAMs or pharmacologic inhibition of Nrp1 signaling causes anti-tumorigenic GAMs to infiltrate and accumulate at the actively expanding glioma border. This is correlated with a reduction in overall tumor vascularity and growth. While it is possible that some macrophages did not undergo cre-recombinase induced excision of the Nrp1 gene, a large population of Nrp1-depleted-, CSF1R-expressing cells was apparent in tumors of Nrp1^MgKO^ mice (Figure 2). Our finding that macrophage density within tumors increases when Nrp1 is knocked out is similar to the report of Casazza and colleagues who observed increased infiltration of squamous cell carcinoma tumors by macrophages in mice with Nrp1 depletion of Ly6C expressing myeloid cells [8].

GAMs near the invasive edge of gliomas in humans and mice upregulate expression of TGF-β, which in turn increases glioma invasiveness [38]. Nrp1 functions as co-receptor for TGFβ that amplifies SMAD2/3 signaling in GAMs, which promotes M2 polarization of microglia [19, 25]. We show here that Nrp1^MgKO^ microglia fail to activate SMAD2/3 signaling in response to glioma tumor cell-conditioned media that contains TGFβ in addition to other cytokines, and that this altered combination of signaling pathways underlies their increased M1, anti-tumoral polarization. It should be noted that other Nrp1-mediated pathways, particularly VEGFA, semaphorin 3A, and HGF signaling may also play important roles in the overall modalities of how GAMs associate with the tumor microenvironment. Furthermore, it would be worthwhile to analyze transcriptional differences between wild type and Nrp1^MgKO^ GAMs isolated from tumors to assess significant alterations in gene expression in an unbiased manner.

GL261 tumors have been shown to recapitulate many characteristics of GBM [39]. Evaluation of tumors reveals histologic pleomorphism, pseudopalisades around necrotic areas, and tumoral neoangiogenesis. GL261 tumors are invasive, but not metastatic. Moreover they present moderate immunogenicity. Validation of our results with additional astrocytoma cell lines of both murine and human origin would be important, although to study this pathway with human-derived glioma cell lines, immunocompromised animals are required, which presents a significant drawback in the study of anti-tumoral immunity. Additionally, it would be critical to analyze more glioma biopsies to firmly establish a correlation of Nrp1 expression in macrophages with the severity of the disease course.

Our results are the first to our knowledge to demonstrate that *in vivo* pharmacological inhibition of Nrp1, not just its genetic ablation, results in better outcomes in the glioma disease course. Taken together, these findings support the investigation of Nrp1-selective inhibitors as potential therapeutics for gliomas.

## MATERIALS AND METHODS

### Evaluation of human tumors

Four patients with primary glioma who had undergone surgical resection from 2000 to 2013 at the Stony Brook University Medical Center (Stony Brook, NY) were selected based on the availability of sufficient remnant tissue to support the aims of this study. Patient clinical information was provided by the Cancer Registry of Stony Brook University Medical Center, including data about patient age, sex, treatment, tumor recurrence, and survival. Use of archival surgical pathology specimens for immunohistochemical studies (Committees on Research Involving Human Subjects protocol 94651) was approved by the Institution Review Board of Stony Brook University Medical Center. Representative tissue blocks from each case were assembled from the archival collections from Department of Pathology and were graded according to World Health Organization standards, as pilocytic (grade I), grade II, grade III, and grade IV tumors.

Five-micrometer, formalin-fixed, paraffin-embedded tissue sections were deparaffinized in xylenes and rehydrated in graded alcohols. Antigen retrieval was performed in citrate buffer (20 mmol/L, pH 6.0) at 100°C for 20 min. The slides were allowed to dry, after which they were subjected to three PBS washes. Non-specific protein interactions were blocked by pretreatment with 5% goat serum in 0.1% Tween-20-supplement PBS for 1 hour and immunolocalization was performed using anti-human Nrp1 (1:50) (Santa Cruz Technologies) and Iba1 (1:1000) (Wako) primary antibodies. The slides were next washed thrice with PBS, stained with alexa-fluor conjugated secondary antibodies for 2 hours at room temperature, washed with PBS, and cover-slipped with DAPI fluoromount. Co-localization of Nrp1 and iba1 was assessed by confocal microscopy.

### Glioma-derived cell line

The GL261 cell line (available through the Division of Cancer Treatment and Diagnosis Tumor Repository at the National Cancer Institute) is derived from a chemically-induced C57BL/6 murine astroglioma [28]. We previously stably transfected the cells with an eGFP construct, generating the cell line, GL261-eGFP, which has been characterized in culture and in an *in vivo* glioma model [29]. This cell line was expanded in Dulbecco's Modified Eagle Media (DMEM) supplemented with 10% heat-inactivated Fetal Bovine Serum (FBS) and 1x Penicillin/Streptomycin (P/S).

### Animals

Mice were bred under maximum isolation conditions on a 12:12 hour light: dark cycle with food *ad libitum*. Littermates from Nrp1^fl/fl^ x Nrp1^fl/fl^ Csf1R-cre breeding pairs were used for experiments [30, 31]. Mice expressing cre recombinase (Nrp1^MgKO^) effectively lack Nrp1 in microglia and macrophages. Littermates lacking cre recombinase are considered ‘wild-type’. All mice were bred on the C57BL/6 background.

### Genotyping

Tissue was acquired from mice by tail snip. Crude DNA was extracted by boiling the tissue in 50mM NaOH for 10 minutes followed by pH neutralization via the addition of 1M Tris-HCl (pH 8.0). Presence of Csf1R-cre and/or the floxed Nrp1 alleles was confirmed using Crimson Taq PCR (New England Biotechnologies) with primers designed against their appropriate gene sequences (See Supplementary Table 1). All mice were genotyped prior to use.

### Primary microglia

Primary microglia were harvested as previously described [32]. Briefly, 0-3 day old (d0-3) mice were decapitated and their cerebral cortices were dissected in ice-cold 1x Hank's Buffered Salt Solution (1x HBSS, Thermo Fisher Scientific Inc.). The cortices were trypsinized, homogenized, and plated in DMEM (Thermo Fisher Scientific Inc.) supplemented with 10% FBS, and 1x P/S. Media was replaced on the 3^rd^ day. After 10-15 days, the cultures were treated with lidocaine (Sigma), gently rocked, and microglia were isolated from the supernatant, counted, and resuspended in DMEM supplemented with 1%FBS and 1x P/S. For live imaging of microglia, 20μg/mL Mini-Ruby (Invitrogen) was added to media for 24 hours, after which the media was removed, the culture was rinsed in 1x HBSS, and media was replaced [33]. All microglia were used for experiments within 48 hours of plating.

### Evaluation of EG00229 cytotoxicity

For evaluation of EG00229-induced cell death, 10,000 GL261-eGFP cells were plated per well in a 96-well plate and incubated in different concentrations of EG00229 for 24 hours. GL261-eGFP cells were then washed in HBSS, incubated with 1μg/mL propidium iodide for 10 minutes, washed, and fixed in 4%w/v PFA. 63x images were taken in six random fields of view and PI+-stained cells were counted to determine drug-induced cell death.

### Transwell migration assays

GL261-eGFP cells were serum starved for 24 hours then harvested, counted, washed, and resuspended in serum-free DMEM. 10,000 GL261-eGFP cells were plated to 8.0μm transwell inserts (Corning) suspended over DMEM supplemented with 10%FBS containing different concentrations of the Nrp1 inhibitor EG00229. Transwell cultures were allowed to grow for 24 hours after which non-migratory cells were gently scraped from the top of the transwell membranes using cotton Q-tips. Membranes were then fixed in 4%PFA, washed in 1x PBS, stained with DAPI, and cell migration across the membranes evaluated by counting the number of nuclei within five random 20x fields for each transwell insert.

### RNA extraction

RNA was harvested from cells by first removing their media, washing them with PBS, and lysing them with RNA-Bee (Tel-Test) according to the manufacturer's protocol. To obtain cDNA, one microgram of RNA was reverse transcribed on a Veriti Thermocycler (Applied Biosystems) using the High Capacity cDNA Reverse Transcription kit. Amplification of cDNA products was performed using primers against RNA products of interest using a Veriti Thermocycler (see Supplementary Table 1).

### Murine glioma model

Gliomas were established in 3-4 month old mice as previously described [29]. Briefly, mice were anesthetized using atropine (0.6 mg/kg, i.p.) and 2.5% avertin (0.02 mg/g, i.p.). A midline incision was made on the scalp and a small burr hole was drilled in the skull at stereotactic coordinates of bregma, −1 mm anteroposterior and +2 mm mediolateral. 3 × 10^4^ GL261-eGFP cells suspended in 1μL of PBS were injected in anesthetized mice over two minutes at a depth of 3mm. Local delivery of drugs to the gliomas was achieved using mini-osmotic pumps (Alzet) connected via rubber tubing to 3mm cannulas (PlasticsOne) positioned directly over the injection tracks. Pumps were either filled with saline + 0.75%DMSO vehicle or 75μM EG00229 (in 0.75% DMSO) and subcutaneously implanted into the backs of mice, as previously described [29]. After completion of the procedure, the incision was sutured and the mice were placed on a heated surface until fully recovered from anesthesia. Buprenorphine was administered to mice for pain management if signs of distress were observed. If mice were found to have lost more than 30% of their initial body weight over the disease course, they were euthanized. All animal procedures were approved by the Stony Brook University Institutional Animal Care and Use Committee (IACUC).

### Isothermal titration calorimetry

Isothermal titration calorimetry (ITC) experiments were carried out in a reaction buffer (20 mM Tris pH 7.9, 50 mM NaCl) using a VP-ITC titration calorimeter (MicroCal, LLC) with a reaction cell volume of 1.4 mL at 15°C. Recombinant Nrp1b1 and Nrp2b1 were prepared as previously described [26]. Prior to the experiments, Nrp1b1 and Nrp2b1 samples were each dialyzed in the same buffer and all solutions, including the buffer that was used for heat dilution measurements, and were degassed and filtered just before loading into the calorimeter. 5 μM Nrp2b1 or 5 μM Nrp1b1 in the reaction cell was titrated with the 50 μM stock solutions of EG00229. 19 consecutive compound injections of 15 μL at 2 second/μL were applied at 4 minutes intervals, while stirring the reaction solution at 300 rpm constant speed. For heat dilution of the protein, 1.4 mL of reaction buffer in the reaction cell was titrated by 300 μL of 50 μM compound and this value was subtracted from the measured heats of binding. Protein concentrations of the samples used in these experiments were estimated by UV absorbance measurements at 280nm.

### Imaging

Image acquisition was performed on either a Nikon Eclipse E600 Microscope (Nikon) or a Zeiss LSM 510 confocal microscope (Zeiss).

### Immunohistochemistry

For evaluation of tumor tissue at different time points, mice were deeply anesthetized with 2.5% avertin and transcardially perfused with 50 mL PBS followed by 50 mL 4% paraformaldehyde (PFA) in PBS. Brain tissue was removed and post-fixed in 4% PFA/PBS for 12 hours followed by 30%w/v sucrose in PBS at 4°C until fully dehydrated. The brains were then embedded in optimal cutting temperature compound (Tissue-Tek), and sectioned using a Leica cryostat (Nussloch, Germany). 20-μm thick coronal sections of throughout all tumor containing tissue were taken for analysis.

For evaluation of tumor volumes, serial sections were evaluated through the largest portions of tumor and tumor diameters were measured and averaged to calculate an average tumor radius using NIS-Elements software (Nikon Instruments Inc.) As GL261-based tumors grow spherically, volume was calculated using the equation V = 4/3πr^3^.

For evaluation of tumor vascularity, serial sections were stained with anti-CD-31 (1:500) (Millipore). Photomicrographs, including 10 random 40x images within tumors were captured and tumor vascularity for each specimen calculated by summating the total luminal area bounded by CD31+ staining for blood vessels using NIS-elements software (Nikon Instruments Inc.).

For evaluation of intra-tumoral inflammation, sections were stained for either F4/80 (1:500) (Abcam) or iba1 (1:1000) (Wako) to evaluate GAM populations. Areas surrounding the needle tract from the tumor cell injection were excluded from imaging analysis. Random, 63x fields of view within the center of tumors or within the border of tumors were used to evaluate GAM density. Co-staining with iba1 or F4/80 and CD86 (1:50) (Millipore), 1:50 CD206 (R&D Systems Inc.), and 1:500 TSPO (Abcam) was used to evaluate GAM polarization. Additionally, sections were stained with 1:100 Csf1R (VWR) and 1:100 Nrp1 (R&D Biosystems) antibodies. random, 63x images were taken for each section and Image J (NIH) used to calculate Mander's co-localization coefficient between iba1 or F4/80 and each inflammation marker. Ratios of average CD86 to CD206 co-localization were calculated for each specimen to determine the M1/M2 GAM polarization ratio.

### Immunoblotting

Cells or tissue were lysed on ice in 50 mM Tris-HCl (pH 7.4) containing 1% Nonidet P-40, 0.25% Na deoxycholate, 150 mM NaCl, 1% SDS, and 1x protease inhibitor cocktail (Sigma-Aldrich). Debris was spun out of samples at 14,000rpm and tissue supernatant was resuspended and denatured by boiling and treatment with β-mercaptoethanol, run using SDS-PAGE, and transferred to PVDF membranes (Immobilon; Millipore). Membranes were then washed in Tris-buffered saline containing 0.05% tween-20 (pH 7.40, TBS-T), blocked in 5% BSA or 5% skim milk powder for 1 hour and probed using p-Smad2/3 (Cell Signaling, 1:1000), Smad2/3 (Cell Signaling, 1:1000), F4/80 (Abcam, 1:500), Csf1R (VWR, 1:200), Iba1 (WAKO, 1:1000), α-actin (Santa Cruz, 1:5000) or TSPO (Abcam, 1:500) overnight. Membranes were then rinsed in TBS-T, probed with appropriate HRP-conjugated secondary antibodies, rinsed in TBS-T, and exposed to Pierce ECL substrate (Thermo Fisher Scientific Inc.) for 1 minute after which X-ray films (Denville Scientific Inc.) were developed from the membranes. Densitometry analysis was performed using Image J (NIH). Antibodies were stripped from membranes in 0.1% SDS, 1% Tween-20, 1.5% glycine, pH 2.2 stripping buffer (Abcam) via two 10-minute incubations at room temperature.

### Flow cytometry

Mice were heavily anesthetized with avertin and transcardially perfused with 50mL ice cold PBS. Mouse brains were extracted and tumor tissue was micro-dissected and placed in ice cold FACS buffer (pH 7.40, 0.1M PBS, 1mM EDTA, 1% BSA, 50U/mL DNase I). Tissue was then subjected to digestion in papain supplemented with 50U/mL DNase I for 30 minutes followed by gentle trituration. Tissue homogenates were passed through 40μM filters and resuspended in 30% Percoll in 1x HBSS. Samples were spun for 15 minutes at 1500rpm after which the pelleted fractions were resuspended in FACS buffer. Samples were washed and stained with CD11B-APC, CD86-PE, and CD206-Pacific Blue antibodies for 1 hour (all at 1:100, Biolegend). Samples were then washed in FACS buffer thrice, fixed in 1%PFA w/v and cell staining was analyzed on a BD FACS Calibur using BD FACSDiva software (BD Biosciences).

### Statistical analysis

Data comparing two population means were analyzed using Student's T-tests. Data assessing differences between multiple population means were compared by one-way ANOVA with Tukey's post-hoc test analysis. A *p*-value < 0.05 was accepted as designating significant difference between two population means with 95% confidence. Differences in survival between populations were analyzed by the Mantel-Cox log rank test. Statistical analysis was performed using Graphpad Prism (Graphpad Software Inc., La Jolla, CA).

## SUPPLEMENTARY METHODS TABLE AND FIGURES


