# Supplementary material for: Ablation of Neuropilin 1 from glioma-associated microglia and macrophages slows tumor progression
Source: Oncotarget. 2016 Jan 10;7(9):9801–14. doi: 10.18632/oncotarget.6877 (PMC4891085; doi:10.18632/oncotarget.6877)
Supplement: Supplementary file 1 [file oncotarget-07-09801-s001.pdf]

Ablation of neuropilin 1 from glioma-associated microglia and macrophages slows tumor progression

Supplementary Methods

Pharmacokinetics of EG00229 in mice.

Sample Preparation

Mice (n=3 per time-point) were given single intravenous doses of EG00229 at 2.5mg/Kg (Animal Groups 1, 2 and 3). Blood samples were taken at 5, 15, 30, 45, 60, 120, 240 and 480 minutes post-dose. Plasma was prepared and, frozen immediately. All analytical standards were prepared following precipitation of plasma proteins using 20% TFA extraction solution. Study samples were dispensed onto 20% TFA immediately upon thawing to minimise potential degradation of compound. The samples were vortex-mixed and centrifuged to precipitate plasma proteins. Supernatant was removed and analysed using the methods detailed.

Instrument parameters

A Micromass Quattro Micro (S/N:QAA668) with the following ESI source settings was used for this study.

Capillary voltage (kV)	3.75
Sample cone voltage (V)	40
Extractor cone voltage (V)	4
RF lens (V)	0.4
Source temp (°C)	120
Desolvation gas temp (°C)	250
Desolvation gas flow (l/h)	350
Cone gas flow (l/h)	100

Experimental tune parameters

Multiple Reaction Monitoring (MRM) methods were developed with the following parameters.

Compound	Molecular Ion	Transition	Sample cone (V)	Collision Energy (eV)
EG00229	[M+H]	498.1 > 260	40	35
Acebutolol	M+H]	337.5 >115.9	34	22

Chromatography

**Column**	**Thermo Hypersil-Keystone Betabasic C18**	
	100 x 4.6mm 5ìm	
Flow rate	1.00 ml/min	
Injection volume	20µl (EG00254) 30µl (EG00229)	
Mobile phase	A: 100% H2O + 0. 1% TFA	
	B: 60% Acetonitrile / 40% H2O + 0.1% TFA	
Gradient profile	0.00 min	10% B

	6.00 min	40% B
	6.10 min	97% B
	9.00 min	97% B
	9.10 min	10% B
Total Runtime	12 min	
EG00229 RT	6.72 min	
Acebutolol RT	4.86 min	

The limits of detection (LOD) and quantification (LOQ) were determined for EG00229 using the chromatographic gradient listed above. EG00229 was found to have an LOD of ~100ng/ml and the LOQ was set at 250ng/ml.

Intravenous data for EG00229 following a single (2.5mg/Kg) dose All values in ng/ml.

LOQ indicates a value below the limit of quantification (250ng/ml).

Time (min)	Group 1	Group 2	Group 3	Mean
5	18650	19520	21020	19730
15	9965	11320	11750	11012
30	5673	5263	12750	7895
45	4507	5323	3571	4467
60	5826	1992	3445	3754
120	1138	997	1079	1071
240	LOQ	LOQ	LOQ	LOQ
480	LOQ	LOQ	LOQ	LOQ

Supplementary Table 1
Sequences of primers used for detecting Nrp1 mRNA, CSF1R-cre DNA, and GAPDH mRNA.

Supplementary Table 1

Gene	Forward Primer	Reverse Primer
Nrp1	GGGCAGAGACTGCAAGTATGA	AGAAATGGCCCTGAAGACAC
CSF1R-cre	CAGGGCCTTCTCCACACCAGC	CTGGCTGTGAAGACCATC
GAPDH	GCACAGTCAAGGCCGAGAAT	GCCTTCTCCATGGTGGTGAA

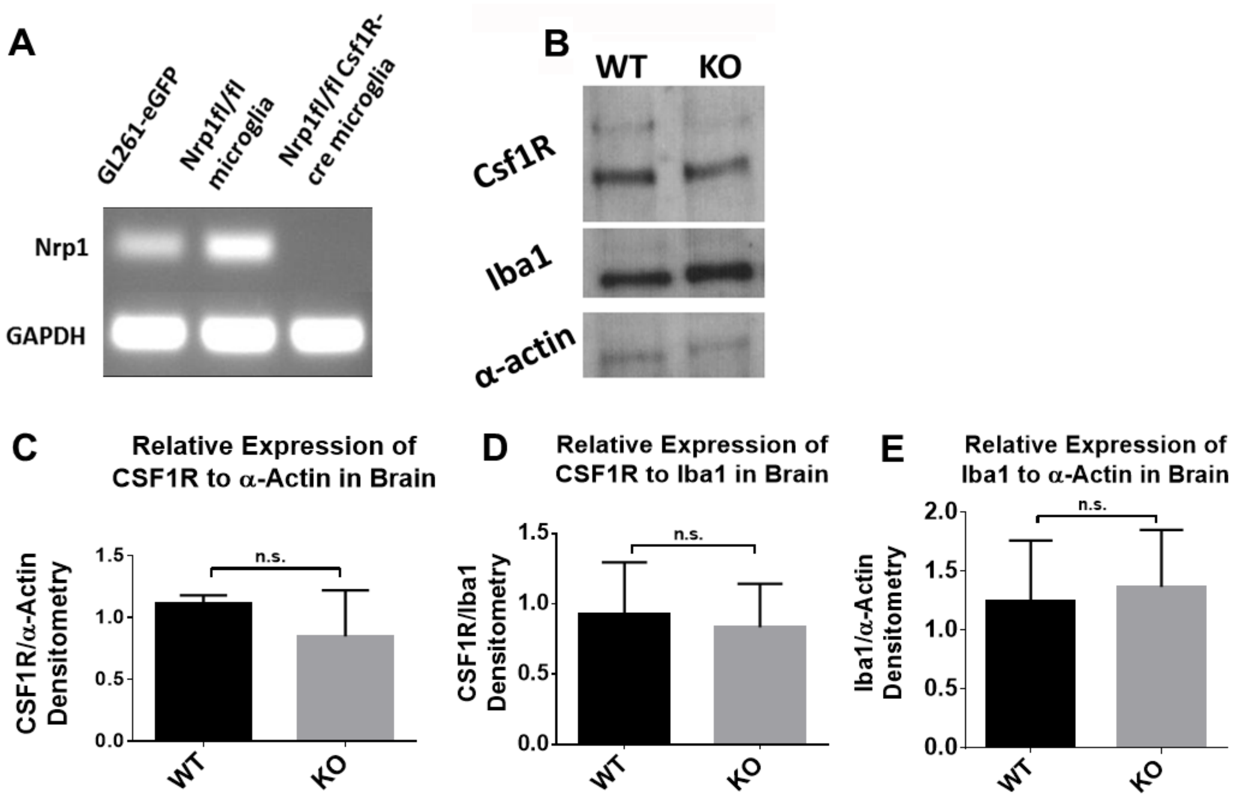

Supplementary Figure 1

A. Expression of Nrp1 mRNA and GAPDH mRNA (loading control) from GL261-eGFP cells, Nrp1$^{fl/fl}$ (wt) microglia, and Nrp1$^{fl/fl}$ Csf1R-cre (Nrp1^{MgKO}) microglia. **B.** Representative blot of Csf1R, Iba1, and α-actin expression in wt and Nrp1^{MgKO} brain. **C-E.** Ratios of Csf1R to α-actin (**C**), Csf1R to Iba1 (**D**), and Iba1 to α-actin expression (**E**) from whole brain lysate in wt and Nrp1^{MgKO} mice (n.s. = $p>0.05$).

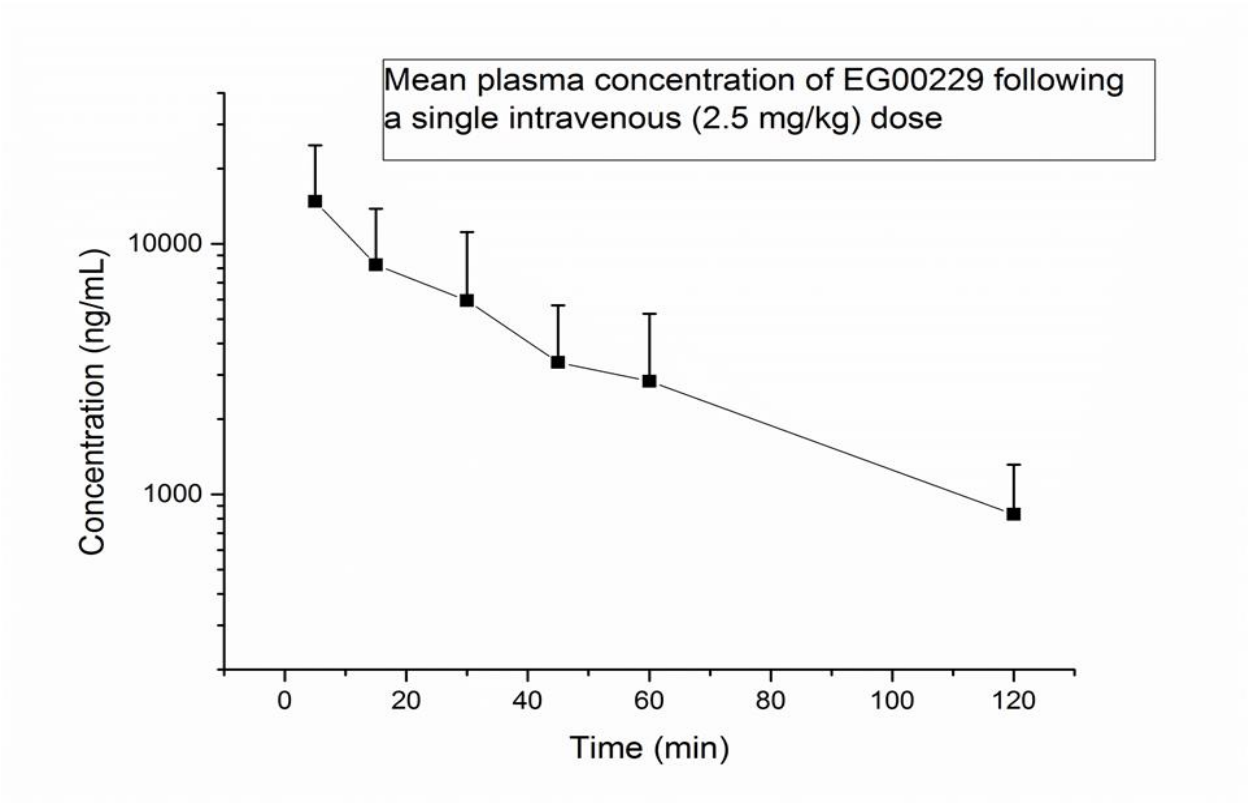

Supplementary Figure 2
Evaluation of intravenous EG00229 administration in mice. **A.** Mean plasma concentration and **B.** pharmacokinetic data for EG00229 in mice which received a single, intravenous 2.5mg/kg dose.

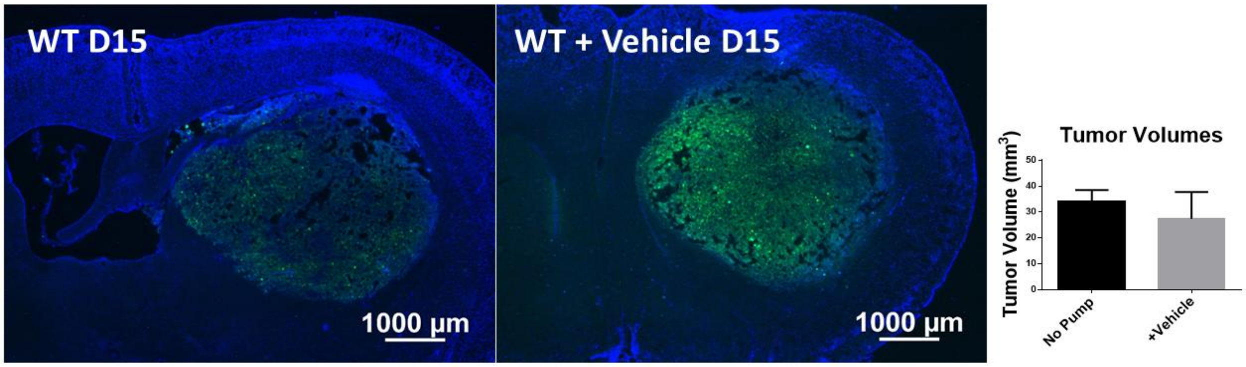

Supplementary Figure 3
Comparison of glioma development in wt mice with no cannula (No Pump) at 15 days vs wt mice treated with saline vehicle +1%DMSO via mini-osmotic pump (+Vehicle) for 15 days. (No Pump: n=5, Vehicle: n=4, Blue =DAPI, Green = eGFP)

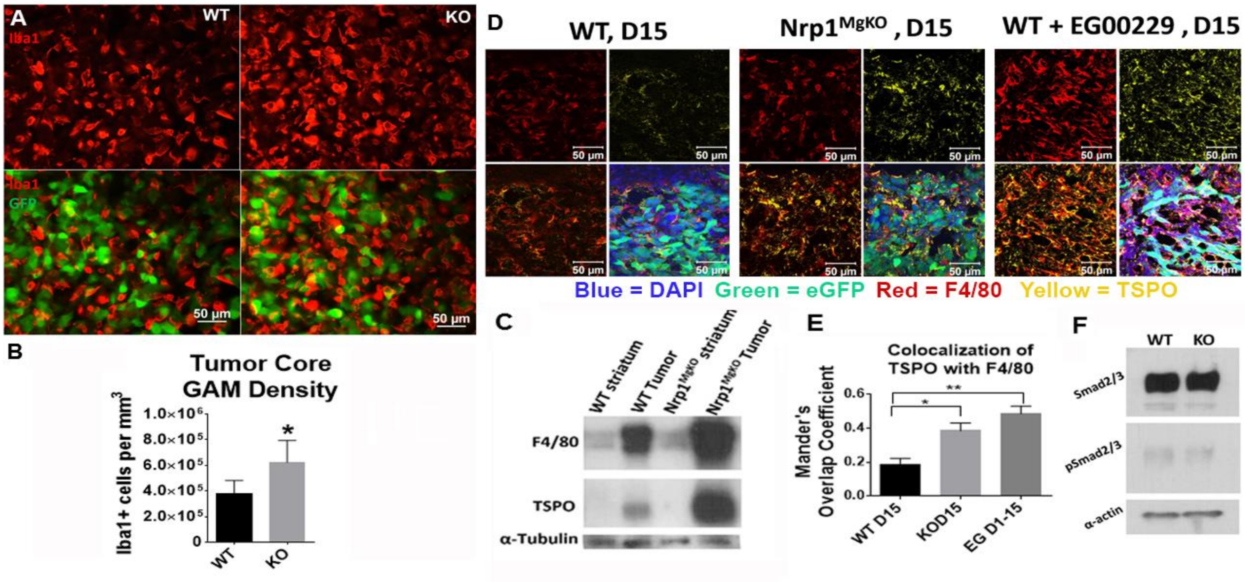

Supplementary Figure 4

A. Representative staining and **B.** quantification of Iba1+ cells within the central core of tumors in wt and Nrp1^{MgKO} mice 15 days PGI (WT: n=6, KO: n=6, *=P<0.05) **C.** Expression of F4/80 (a microglial/macrophage marker), TSPO (a marker of microglial activation), and α-tubulin from crude tumor or normal striatum from a wt and Nrp1^{MgKO} mouse. **D.** Representative images of the expression of TSPO (yellow) in F4/80 labelled GAMs (red) within the tumor rims of wild type (WT), Nrp1^{MgKO} (KO), and EG00229-treated wt mice (EG00229) at 15 days post disease induction. Tumor cells express GFP (green). **E**. Quantification of the colocalization of TSPO and F4/80 in the tumor rims of gliomas from wt, Nrp1^{MgKO}, and EG00229-treated mice (*=p<.05, **=p<0.01; WT D15: n=5; KO D15: n=6; EG D1-15). **F.** Blot comparing the expression of pSMAD2/3, SMAD2/3, and α-actin expression between wt and Nrp1^{MgKO} microglia.

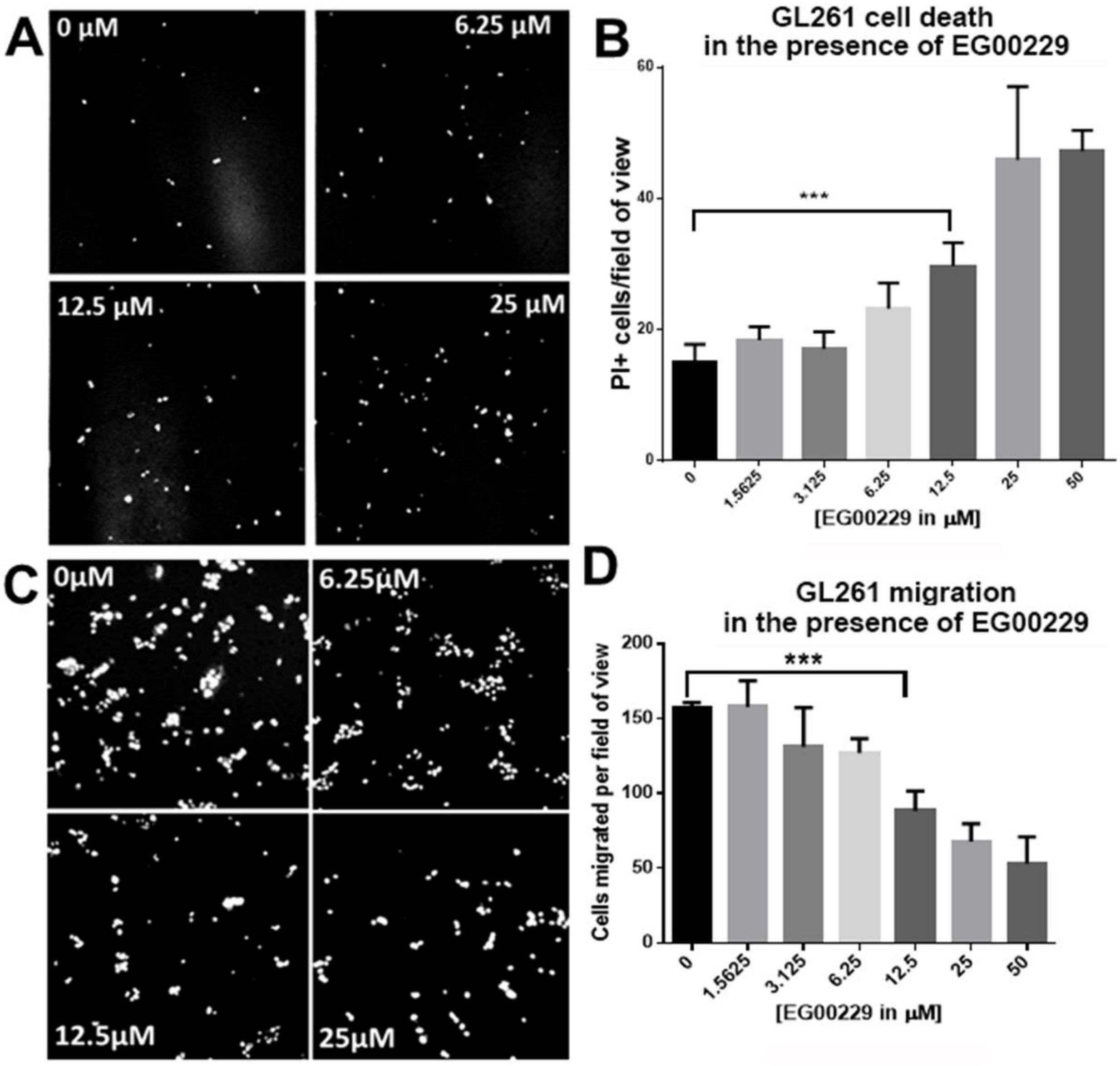

Supplementary Figure 5

A. Representative images of propidium iodide (PI) staining of GL261-eGFP cells incubated with increasing concentrations of EG00229 for 12 hours. **B**. Quantification of average number of PI^+ cells per 40x field of view with increasing EG00229 concentrations. **C-D**. Representative images (**C**) and quantification (**D)** of the number of DAPI-stained nuclei of GL261-eGFP cells which migrated across transwell membranes towards serum-supplemented media in the presence of increasing EG00229 concentrations (***=p<.001).
